# Flunixin Meglumine Reduces Milk Isoprostane Concentrations in Holstein Dairy Cattle Suffering from Acute Coliform Mastitis

**DOI:** 10.3390/antiox10060834

**Published:** 2021-05-24

**Authors:** Carsten C. F. Walker, Jill L. Brester, Lorraine M. Sordillo

**Affiliations:** Department of Large Animal Clinical Sciences, College of Veterinary Medicine, Michigan State University, East Lansing, MI 48824, USA; walke490@msu.edu (C.C.F.W.); brester@msu.edu (J.L.B.)

**Keywords:** mastitis, coliform mastitis, NSAID, flunixin meglumine, redox balance, oxidant status, inflammation, COX-2, dairy cattle

## Abstract

Dysfunctional inflammation contributes significantly to the pathogenesis of coliform mastitis and the classical pro-inflammatory enzyme cyclooxygenase-2 (COX-2) is the target of medical intervention using the non-steroidal anti-inflammatory drug (NSAID) flunixin meglumine (FM). Inhibition of COX-2 by FM can decrease concentrations of pro-inflammatory fatty acid-based mediators called eicosanoids, providing antipyretic and analgesic effects in dairy cows suffering from coliform mastitis. However, approximately 50% of naturally occurring coliform mastitis with systemic involvement results in death of the animal, even with NSAID treatment. Inadequate antioxidant potential (AOP) to neutralize reactive oxygen species (ROS) produced during excessive inflammation allows for oxidative stress (OS), contributing to tissue damage during coliform mastitis. Biomarkers of lipid peroxidation by ROS, called isoprostanes (IsoP), were used in humans and cattle to quantify the extent of OS. Blood IsoP were shown to be elevated and correlate with oxidant status during acute coliform mastitis. However, the effect of FM treatment on oxidant status and markers of OS has not been established. Blood IsoP concentrations were used to quantify systemic OS, whereas milk was used to assess local OS in the mammary gland. Results indicate that FM treatment had no effect on blood markers of inflammation but reduced the oxidant status index (OSi) by increasing blood AOP from pre- to post-FM treatment. Milk AOP significantly increased from pre- to post-FM treatment, whereas ROS decreased, resulting in a decreased OSi from pre- to post-FM treatment. The only blood IsoP concentration that was significantly different was 5-iso-iPF2α-VI, with a decreased concentration from pre- to post-FM treatment. Conversely, milk 5-iso-iPF2α-VI, 8,12-iso-iPF2α-VI, and total IsoP concentrations were decreased following FM treatment. These results indicated that administration of FM did improve systemic and local oxidant status and reduced local markers of OS. However, differential effects were observed between those animals that survived the infection and those that died, indicating that pre-existing inflammation and oxidant status greatly affect efficacy of FM and may be the key to reducing severity and mortality associated with acute coliform infections. Supplementation to improve AOP and anti-inflammatory mediator production may significantly improve efficacy of FM treatment.

## 1. Introduction

Mastitis is the costliest infectious disease in the US dairy industry, with over $2 billion in annual deficits due to loss of milk production and potential death of the animal [1,2]. Particularly problematic in dairy cows are coliform mastitis cases that are the result of *Escherichia coli* (*E. coli*) [2,3,4,5,6]. Targeted breakdown of the mammary epithelium during coliform mastitis results in decline of milk quality and production [7,8,9,10,11,12,13,14]. Some animals mount an effective inflammatory response to clear the pathogen without clinical signs and quickly restore the quality and quantity of milk. However, acute coliform infections can cause dysfunctional inflammation and excessive tissue damage, leading to significant and sometimes permanent cessation of milk production from affected mammary glands. Acute cases of coliform mastitis with systemic involvement may also cause death of infected animals [1,2,3,4,5,6].

An effective immune response is highly dependent on properly regulated inflammation, with rapid onset and timely resolution [10,12]. Stimulation of the Toll-like receptor 4 (TLR4) by the endotoxin lipopolysaccharide (LPS) results in the synthesis of proinflammatory mediators such as tumor necrosis factor alpha (TNFα), interleukin-6 (IL-6), and neutrophil adhesion molecules [15,16,17,18,19,20]. Crucially, TLR4 activation by LPS induces the classical pro-inflammatory enzyme cyclooxygenase-2 (COX-2), increasing the production of fatty acid-derived pro-inflammatory lipid mediators called eicosanoids that include prostaglandins, leukotrienes, and thromboxanes [9,12,13,20]. Moreover, induction of COX-2 is amplified by TNFα, creating a feedback loop [8,10,20], resulting in elevated eicosanoid concentrations that induce IL-6 production by macrophages and neutrophils [20,21,22]. During inflammation, mediators not only act on localized mammary tissues, but also can have systemic effects, leading to increased mobilization of adipose tissue and increased circulating non-esterified fatty acids (NEFAs) [6,13,23,24,25,26,27]. Prolonged elevated NEFAs results in systemically increased production of cytokines, adhesion molecules, eicosanoids through increased expression of COX-2 and free radicals, such as reactive oxygen species (ROS) [28,29,30]. 

Even though ROS are essential to cell signaling and phagocytotic removal of bacteria by neutrophils, lack of adequate antioxidant potential (AOP) to neutralize ROS can be detrimental [31,32,33]. Excessive ROS causes oxidative stress (OS), which can further increase inflammation, as well as the likelihood of redox signaling-induced cell death [11,13,23,24,25,26,31,32,33,34,35]. During OS, ROS damage cellular lipids, proteins, and DNA, the products of which are used as biomarkers to quantify OS. Lipids are particularly susceptible to ROS damage during OS and products of non-enzymatic peroxidation of arachidonic acid (AA) called isoprostanes (IsoP) are commonly measured to quantify the extent of OS in dairy cattle and humans [32,34,35,36,37,38]. In dairy cattle, plasma IsoP concentrations are elevated during naturally occurring coliform mastitis and correlate with systemic oxidant status [25]. Additionally, milk IsoP concentrations are correlated with inflammation and represent lipid peroxidation events of the mammary gland rather than systemic oxidant status [25,26]. Not only does OS result in tissue damage, but it also further induces the inflammatory response, increasing the likelihood of disease progression with systemic involvement [11,12,13,24,25,31,32,33,34,35,36]. 

A key feature of acute coliform mastitis is excessive eicosanoid synthesis that leads to many systemic symptoms such as pyrexia, nociceptive, and inflammatory pain [11,12,13,14,15,20,24,29]. Focused research on eicosanoid biosynthesis and activity has led to FDA approval for the administration of the non-steroidal anti-inflammatory drug (NSAID) flunixin meglumine (FM) to animals suffering from endotoxemia associated with acute coliform mastitis [39]. Competitive inhibition of the COX-2 enzyme by FM reduces eicosanoid concentrations observed in naturally occurring and experimentally induced systemic coliform mastitis, resulting in reduced rectal temperature and analgesic effects [1,2,40,41,42]. However, efficacy of FM treatment has not been optimized, as approximately half of all dairy cows suffering from acute coliform mastitis succumb to the infection [4,5]. Increased interest in the underlying mechanism of the conflicting reports on efficacy of FM treatment has been focused on classic markers of inflammation pre- and post-FM treatment. For example, previous studies found that ex vivo COX-2 mRNA expression in whole blood of dairy cows is not altered eight hours post-FM treatment, whereas TNFα mRNA expression was significantly reduced [43,44]. Although OS plays a central role in the pathogenesis of coliform mastitis, there is no information available on how oxidant status is affected in animals treated with FM for endotoxemia associated with acute coliform mastitis. 

The effect of FM on the oxidant status and markers of oxidative stress may hold answers to the underlying mechanisms resulting in the seemingly equivocal survival of animals suffering from naturally occurring acute coliform mastitis. Therefore, the goal of this study was to establish a time-course of blood and milk biomarkers of inflammation, oxidant status, and OS for animals suffering from naturally occurring systemic coliform mastitis treated with FM. We hypothesize that FM treatment will reduce the OSi and IsoP concentrations in dairy cows suffering from endotoxemia associated with acute coliform mastitis. 

## 2. Materials and Methods

### 2.1. Animal Selection and Study Design

All animals in this study were Holstein dairy cattle housed at a commercial dairy in mid-Michigan. A total of 16 animals, 8 with acute coliform mastitis and 8 matched healthy control animals, were used in this study. Animals were enrolled in this study when suffering from acute coliform mastitis with systemic involvement and on-farm protocol dictated that veterinary care, including intravenous treatment with the NSAID flunixin meglumine, was necessary. Cows had to have positive *E. coli* milk cultures (>100 colony forming units) and at least 2 signs of systemic clinical disease. Signs of acute coliform mastitis included increased core body temperature (>39.2 °C), tachycardia (heart rate > 80 beats/minute), tachypnea (respiratory rate > 30 breaths/minute), episcleral injection, local signs of mammary gland inflammation including discoloration, swelling, heat and pain on palpation, and typical serum-like watery milk as determined by the herd veterinarian. Initial blood and milk samples were collected immediately before treatment with a single intravenous dose of flunixin meglumine (2.2 mg/kg IV), ceftiofur sodium (2.2 mg/kg SC), and oral electrolyte fluids according to standard farm treatment protocols (pre-FM treatment); 8 h post-treatment (post-FM treatment); and again, immediately before the animal succumbed to the infection or recovered and was returned to the milking herd or sold (Resolution). One animal succumbed to the infection within 4 h of the post-FM treatment sample and no third sample collection was possible. A total of 8 individually matched control (MC) animals were chosen based on the enrolled systemic coliform animals and matched for days in milk (DIM), lactation number (LAC), and phenotype (number of milking quarters). Mean days in milk (DIM) for systemically infected animals was 101.63 (±50.3) and the mean lactation number was 3.125 (±0.687). Animals enrolled in the MC group did not receive FM and had negative bacterial milk cultures, no overt clinical signs, and a somatic cell count of <200,000 cells/mL.

### 2.2. Blood and Milk Sample Collection

Blood samples were collected with Vacutainer tubes containing serum separator and EDTA. Pre-treatment samples of systemic coliform infected animals were collected from the jugular vein, all subsequent blood samples from the same animal were collected from the coccygeal vein. Blood samples from healthy matched control animals were taken from the coccygeal vein and occurred within 14 days of initial pre-treatment sample from the paired systemically infected animal. Blood collection at each sampling point consisted of two 10 mL EDTA tubes and two 15 mL serum separator tubes. After transport on ice, EDTA tubes were treated with 4 uL/mL of antioxidant/reducing agent (ARA) before centrifugation at 1449× *g* for 15 min at 4 °C. Serum separator tubes were kept at room temp until properly coagulated, before being placed on ice and centrifuged at 1449× *g* for 15 min at 4 °C. After processing, 1 mL of serum and plasma was aliquoted into 1.5/2 mL microcentrifuge tubes and snap frozen in liquid nitrogen before being stored at −80 °C. 

Milk samples were obtained at the same time as blood samples were taken and placed on ice. Milk samples were obtained aseptically from all active quarters of the animals, including the infected quarter(s), and pooled. Volume of pooled samples was at least 10 mL. Two 1 mL aliquots of each pooled milk sample were flash frozen without the addition of ARA, the remainder of the pooled sample was treated with 4 uL/mL ARA and then aliquoted and snap frozen in liquid nitrogen before being stored at −80 °C.

### 2.3. Blood and Milk Biomarker Analysis

Blood biomarkers haptoglobin and NEFAs were analyzed in serum using commercially available kits for the small-scale biochemistry analyzer (CataChemWell-T; Catachem Inc.). Serum ROS was carried out using the Oxyselect in vitro kits (Cell BioLabs Inc., San Diego, CA, USA), as previously outlined in Putman et al., 2018 [45]. Briefly, 96-well microtiter plates (Black Isoplate-96, PerkinElmer, Waltham, MA) were read using the Biotek H1 plate reader (Biotek, Winooski, VT, USA). Dichlorofluorescent dye fluorescence was determined at 480 nm of excitation and 530 nm of emission. A standard curve (0–10,000 nM) was created to ensure fluorescence at various concentrations. Background fluorescence was eliminated by subtracting blank values from sample values. Units were measured as relative fluorescence units (RFU) per microliter. Serum AOP was carried out as previously described by Re et al., 1999 [46]. Briefly, the AOP of a sample was determined by the ability to reduce 2,20-azinobis-3-ethylbenzothiazoline-6-sulfonic acid (ABTS) (Sigma-Aldrich, St. Louis, MO, USA) and standardized to the reduction capacity of trolox (synthetic vitamin E analog). Serum amyloid a (SAA) was analyzed using commercially available kits from Tridelta Development Ltd. (Maynooth, Ireland). ELISAs were performed for TNFα and IL-6, using standard bovine ELISA kits from Thermo Fischer Scientific (Waltham, MA, USA). All assays were read out using the Biotek H1 plate reader.

### 2.4. Reagents

Acetonitrile, methanol, and formic acid of liquid chromatography–MS grade were purchased from SigmaAldrich (St. Louis, MO). Deuterated and non-deuterated isoprostane standards were purchased from Cayman Chemical (Ann Arbor, MI, USA). Butylated hydroxy toluene (BHT) was purchased from Acros (Waltham, MA, USA), 156 EDTA and triphenylphosphine were purchased from SigmaAldrich, and indomethacin was purchased from Cayman Chemical.

### 2.5. Solid-Phase Extraction (SPE) of Blood and Milk

Blood and milk samples were extracted and analyzed using methods published previously by Mavangira et al. 2016 [25]. Briefly, milk (4 mL) and plasma (2 mL) samples were mixed with an antioxidant-reducing agent mixture (4 μL of antioxidant-reducing agent/1 mL of sample) to prevent degradation of preformed oxylipids and prevent ex vivo lipid peroxidation as described previously the antioxidant-reducing agent mixture consisted of 50% methanol, 25% ethanol, and 25% water with 0.9 mM of BHT, 0.54 mM EDTA, 3.2 mM TPP, and 5.6 mM indomethacin. 

Plasma extractions were done using an Oasis plasma protocol on an Extrahera robot by Biotage (Charlotte, NC, USA). The samples, including formic acid, ARA, and Internal Standard were loaded into Oasis HLB 12 cc 500 mg LP extraction columns (Waters, Milford, MA, USA) and eluted using a 90:10 acetonitrile (ACN) and methanol (MeOH). Once the samples were eluted, they were evaporated using a Savant SpeedVac, resuspended in 150 µL of 2:1 MeOH and ddH_2_O, filtered through an Amicon Ultrafree-MC filter (Sigma-Aldrich, St. Louis, MO, USA) and transferred to a chromatography vial with insert and stored at −20 °C until LC/MS/MS analysis. 

Milk solid-phase extractions with hydrolysis were done on samples treated with ARA using methods previously described in Mavangira et al., 2015 [47] with modifications. Briefly, 1 mL of whole milk treated with ARA was thawed on ice and treated with an additional 20 µL of ARA before addition if 710 µL of 6 M potassium hydroxide (KOH). After 45 min of incubation at 45 °C the samples were cooled to room temp and 3 mL of acetonitrile with 1% formic acid was added and vortexed. Supernatant was removed after centrifugation at 1976× *g* for 10 min at 4 °C and added to 40 mL of HPLC-grade water. At this time, the internal standard was added and SPE using the Oasis HLB 12 cc 500 mg LP extraction columns as described in Mavangira et al., 2015 [47].

### 2.6. LC/MS/MS Analysis

Details of LC/MS/MS analysis are described in Mavangira et al., 2016 [25]. In short, the quantification of metabolites was accomplished on a Waters Xevo-TQ-S tandem quadrupole mass spectrometer using multiple reaction monitoring (MRM). Chromatography separation was performed with an Ascentis Express C18 HPLC column (10 cm × 2.1 mm; 2.7 μm particles, Sigma-Aldrich, St. Louis, MO, USA) at 50 °C, with the autosampler at 10 °C. Mobile phase A was water containing 0.1% formic acid, and mobile phase B was acetonitrile. Flow rate was fixed at 0.3 mL/ min. Liquid chromatography separation took 15 min per sample. MRM parameters including cone voltage, collision voltage, precursor ion, product ion, and dwell time were optimized based on Waters QuanOptimize software by flow injection of pure standard for each individual compound. Total IsoP concentrations were obtained by addition of IsoP detected in each sample type.

### 2.7. Statistical Analysis

All statistics were calculated using SAS9.4. For pairwise comparison. the Wilcoxon Rank-Sum score was used and means with standard error of the mean (SEM) was reported. Multiple comparisons of timepoints over the course of the infection were done using a two-way ANOVA, with uneven timepoints, repeated measures, and Tukey–Kramer adjustment. Non-normally distributed data were either log10 or square root transformed and back transformed for graphical representation. Multiple comparisons were sliced by time and treatment, for the discrete analysis. 

## 3. Results

### 3.1. Survival Rate

Of the eight animals with acute coliform mastitis that were enrolled in this study, four animals survived. Therefore, an inclusive and discrete analysis of the infection outcome groups were performed. 

### 3.2. Blood

**Figure 1:**
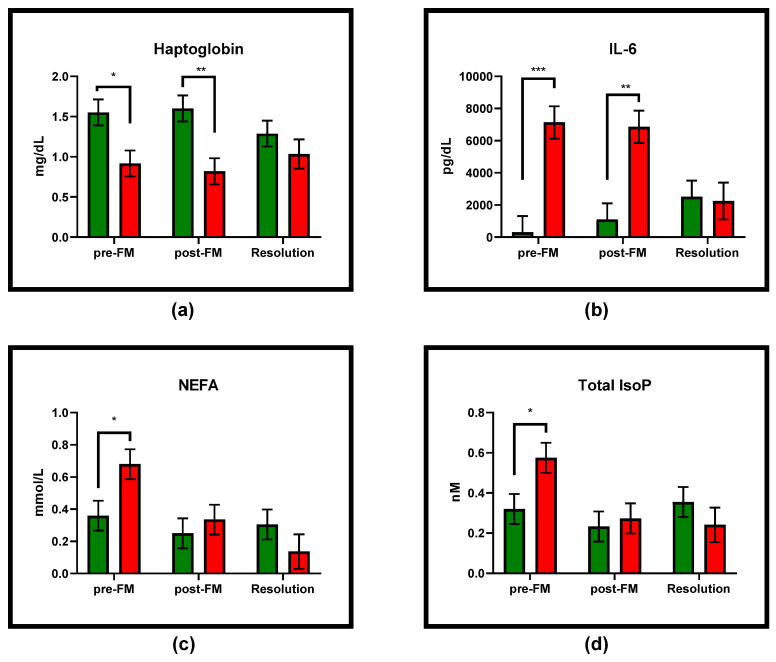
Concentrations (mean ± SEM) of blood biomarkers that differed between the infection outcome groups (green bars = survived; red bars = died) at any of the sampling timepoints: (**a**) haptoglobin was elevated among cows that survived pre- and post-FM treatment, but was not different between outcome groups at resolution; (**b**) IL-6 was elevated pre- and post-FM treatment among cows that died, but was not different between outcome groups at resolution; (**c**) NEFAs were elevated pre-FM treatment among cows that died, but not post-FM treatment or at resolution; (**d**) total IsoP pre-FM treatment were elevated among cows that died, but not post-FM treatment or at resolution. * *p* < 0.05, ** *p* < 0.01, and *** *p* < 0.001.

Eight hours after treatment with FM, no overall change in any of the inflammatory biomarkers analyzed in this study was observed among animals with systemic coliform mastitis. Similarly, there was no change in inflammatory marker concentrations 8 h post-FM treatment, when analyzed by infection outcome. Interestingly, pre-FM treatment concentrations of haptoglobin, IL-6, and NEFAs were significantly different between infection outcome groups (Figure 1). Pre-treatment concentrations of IL-6 and NEFAs were elevated (*p* = 0.0005 and *p* = 0.0334, respectively), whereas haptoglobin concentrations were decreased among those animals that survived the infection, compared to those that did not (*p* = 0.0181). Haptoglobin remained significantly decreased 8 h post-FM treatment among the animals that survived compared to those that did not (*p* = 0.0057) and IL-6 remained elevated (*p* = 0.0019). Even though no change from pre- to post-FM treatment was observed, NEFA concentrations were no longer significantly elevated among animals that died at 8 h post-FM treatment when compared to those that survived (Figure 1). 

Analysis of the overall blood oxidant status revealed a decrease in OSi from pre- to post-FM treatment (*p* = 0.0134) which is most likely driven by an overall increase in AOP post-FM treatment (*p* = 0.0194) (Table 1a). However, when analyzed by infection outcome, no change in AOP from pre- to post-FM treatment was observed in either outcome group. Interestingly, the OSi of the animals that survived was decreased 8 h post-FM treatment (*p* = 0.0094), whereas the OSi of those that died remained unchanged (Table 1b).

Only the IsoPs 5-iso-iPF2α-VI and 8,12-iso-iPF2α-VI were detected in blood and neither had significant changes in concentrations, overall and by infection outcome, from pre- to post-FM treatment. Total blood IsoP concentrations were also not affected by 8 h of FM treatment. However, pre-FM treatment total IsoP concentrations were significantly elevated among animals that died compared to those that survived (*p* = 0.0358) but were no longer different 8 h post-FM treatment. 

### 3.3. Milk

**Figure 2:**
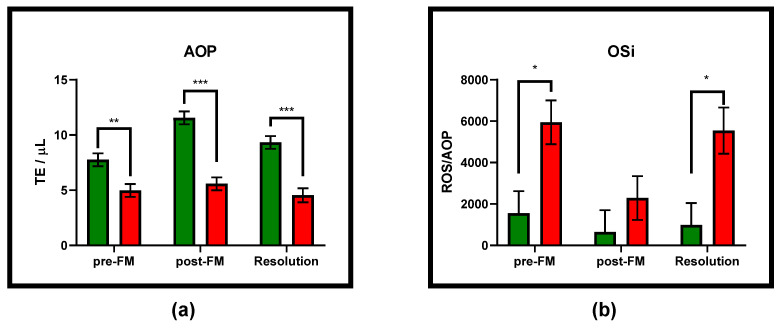
Concentrations (mean ± SEM) of milk biomarkers that differed between the infection outcome groups (green bars = survived; red bars = died) at any of the sampling timepoints: (**a**) AOP was elevated among cows that survived at all three timepoints; (**b**) the OSi was elevated pre-FM treatment and at resolution among cows that died, but not post-FM treatment. * *p* < 0.05, ** *p* < 0.01, and *** *p* < 0.001.

Flunixin meglumine treatment decreased overall ROS concentrations in milk from pre- to 8 h post-FM treatment (*p* = 0.0054), increased AOP (*p* = 0.0051), and lowered the overall OSi (*p* = 0.0131) (Table 2). However, FM treatment only decreased ROS concentration among those animals that died (*p* = 0.0045) and increased AOP only among those that survived (*p* < 0.0001), resulting in a decreased OSi among those animals that died (*p* = 0.0003), but not those that survived. Most strikingly, elevated AOP among animals that survived compared to those that died persisted throughout all three sample times (Figure 2), whereas OSi of the animals that survived is only decreased pre-FM treatment (*p* = 0.0135) but not 8 h post-FM treatment when compared to animals that died. At no timepoint are ROS concentrations different among infection outcome groups. 

In milk, 8-iso-15(R)-PGF2α and 8-iso15-keto-PGE2, as well as 5-iso-iPF2α-VI and 8,12-iso-iPF2α-VI, were detected and contributed to the total IsoP values. Eight-hour treatment with FM decreased overall 5-iso-iPF2α-VI and 8,12-iso-iPF2α-VI concentrations (*p* = 0.0024 and *p* = 0.0039, respectively), as well as the total IsoP concentration (*p* = 0.0017) from pre- to post-treatment (Table 3). Strikingly, the discrete analysis of infection outcome groups revealed that none of the IsoP detected, nor the total IsoP concentration, changed pre- to post-FM treatment in animals that died of the infection. Whereas 5-iso-iPF2α-VI, 8,12-iso-iPF2α-VI, and total IsoP were decreased after at 8 h post-FM treatment (*p* = 0.0064, *p* = 0.0131, and *p* = 0.0084, respectively). However, at no sample time were any of the individual IsoP, or the total IsoP, different from one another.

## 4. Discussion

### 4.1. Traditional Inflammatory Markers

Cytokines and acute-phase proteins such as TNFα, IL-6, and haptoglobin have been used as inflammatory markers during acute coliform mastitis. Elevated TNFα and IL-6 have been associated with the severity of systemic and local inflammation [4,5,6,7,8,10,11,13,48,49], and a reduction in TNFα by FM treatment has been demonstrated in vitro and ex vivo [40,43,44]. However, these classical inflammatory biomarkers may not be accurate particularly when assessed under field conditions with naturally occurring coliform mastitis. The lack of disparity in TNFα concentrations with FM treatment or between infection outcome groups recorded in our study may be due to the timing of sampling, as the duration of the elevated TNFα concentrations differ greatly among varying experimental designs of induced mastitis [8,43,44,48,49,50]. Additionally, elevated IL-6 concentrations among animals that died in this study are in contrast to earlier reports of elevated IL-6 among animals that survived naturally occurring coliform mastitis [4] and the lack of difference in IL-6 concentrations observed among mild and acute infections [5]. Haptoglobin concentrations increase with naturally occurring coliform mastitis [2,25,48], but there was no link between disease severity and haptoglobin concentrations following intramammary challenge with *E. coli* [48]. Prior to this study, no data exist comparing haptoglobin concentrations among animals that survived and died of coliform mastitis. Less serum haptoglobin concentrations among cows that died in this study may be indicative of a prolonged exposure to endotoxin, as repeated experimental LPS infusion (every 24 h) resulted in peak haptoglobin concentrations at 48 h with a reduction within 72 h [49]. However, haptoglobin concentrations were shown to be increased during naturally occurring acute infections with systemic involvement compared to mild cases, reaching peak concentrations 3 days after establishment of systemic involvement and initial sampling [5]. Ultimately, the limited in vivo studies of naturally occurring coliform mastitis that record TNFα, IL-6, and/or haptoglobin provide conflicting results. Attempts to describe classical inflammatory biomarkers with induced coliform mastitis also are conflicting, which may be due to variation in experimental design and the timing of infection with respect to appearance of clinical symptoms. Due to the dynamic nature of the cytokine and acute-phase protein cascades, the use of classical inflammatory markers such as TNFα, IL-6, and haptoglobin may be less effective under field conditions. 

Elevated NEFA concentrations have been linked to severity of inflammation during coliform mastitis [25,47]. Elevated pre-FM treatment circulating NEFAs among cows that died in this study, when compared to those that survived, may be indicative of an exacerbated inflammatory response since circulating NEFAs and COX-2 enzyme can be increased via upregulation of Toll-like receptor (TLR) signaling [21,28,30]. Increased production of pro-inflammatory eicosanoids through the COX pathway can then lead to further adipose mobilization and NEFA release, creating a positive feedback cycle [23,29]. Additionally, relevant is that elevated NEFAs can increase mitochondrial ROS production [35,51] and contribute to the elevated milk ROS seen in this study. Serum NEFA concentrations among the cows that died were no longer elevated compared to the cows that survived post-FM treatment, which can be attributed to a reduction in pro-inflammatory eicosanoids or direct action of FM on adipocytes [52]. 

Additionally, NEFAs are a source of polyunsaturated fatty acids (PUFAs) that serve as substrates for enhanced arachidonic acid (AA) metabolism through the COX pathway, consisting of the inducible COX-2 and constitutively expressed COX-1 enzymes [53]. Metabolism of AA results not only in pro-inflammatory eicosanoids, but also ROS mediators able to damage cellular macromolecules directly and modulate ROS signaling pathways [31,54,55]. Particularly during *E. coli* mastitis, TLR4 activation by LPS and subsequent production of specific eicosanoids responsible for endothelial barrier impairment was documented [10,12,13,15,38]. Lastly, availability of PUFA substrate was shown to greatly influence lipid mediator production through COX-2 in dairy cattle suffering from coliform mastitis, most likely through the interaction of the allosteric and catalytic subunits of the COX isomers [47,53,56,57,58] and shunting of substrates to other enzymatic and non-enzymatic metabolic pathways of fatty acids [47]. 

### 4.2. Oxidant Status and Isoprostanes

This study documented for the first time that IsoP are potentially superior biomarkers over classically used cytokines or acute-phase proteins in establishing the extent of inflammation and efficacy of NSAID treatment in dairy cattle suffering from acute coliform mastitis. As products of ROS-mediated lipid peroxidation, IsoP may present ideal endpoint biomarkers of excessive inflammation. Moderate amounts of ROS are crucial signaling molecules of cellular processes, such as proliferation, metabolic adaption, and differentiation, but excessive accumulation of ROS can cause pro-oxidant damage to cells and tissues. As such, ROS accumulation must be controlled by cellular and systemic AOP to avoid lipid peroxidation and the formation of IsoP. Dysfunctional inflammation associated with acute coliform mastitis results in an imbalance of the oxidant status, OS, and the formation of IsoP [24,32,53]. In this study, treatment with FM decreased 5-iso-iPF2α-VI, 8,12-iso-iPF2α-VI, and total IsoP in milk of the cows that survived, demonstrating a reduction in local OS. Localized OS is a key feature of acute coliform mastitis, as the elevated ROS production by neutrophils and macrophages recruited to the infection site causes significant OS [33,35]. Indeed, even though milk ROS was decreased with FM treatment among cows that died, it did not result in a decreased IsoP production. A possible explanation is that the extent of tissue damage among cows that died prior to FM administration was too extensive for the animal to take advantage of the reported ex vivo improved epithelial barrier integrity of bovine mammary epithelial cells treated with FM [44]. 

The resulting continuous influx of neutrophils and macrophages into infected mammary tissue may have resulted in the sustained IL-6 concentration observed and prevented the AOP to sufficiently neutralize ROS production by macrophages, resulting in sustained milk IsoP production. Lack of milk IsoP reduction among animals that died may also be tied to the lack of milk AOP increase, indicating a depleted antioxidant supply due to possibly prolonged acute local inflammation, lack of adequate feed intake [32] or compromised liver function causing systemic oxidant status changes [53]. However, from this study we cannot determine if decreased AOP among cows that died is a result or cause of excessive inflammation. Nonetheless, elevated pre-FM total plasma IsoP among cows that died compared to those that survived and the improved oxidant status from pre- to post-FM treatment show the potential of IsoP to not only be used as a biomarker to measure the extent of local and systemic inflammation, but to also measure efficacy of medical interventions. Certainly, the inconsistent nature of traditional inflammatory biomarkers such as cytokines and acute-phase proteins highlights the potential of IsoP as an endpoint biomarker for inflammation in dairy cattle, not limited to acute coliform mastitis. Most importantly, quantification of IsoP highlights how defining the pre-existing inflammatory state and associated oxidant status are on the efficacy of FM treatment. Additional studies are needed to assess how local and systemic synthesis of IsoP and their possible bioactivity may impact disease progression. 

Additional work relating to the differential production of specific IsoP may be of value. Limited knowledge exists on the specificity of IsoP production, but preferential production of E2- and D2-IsoP over F2-IsoP with depleted α-tocopherol [59] and variations of IsoP production during mammary gland involution [45], are good indicators of regulated IsoP synthesis. In this study, only F2-IsoP were affected by FM treatment, whereas E2-IsoP did not change in milk and was not detected in blood. We were also not able to detect A- and D-IsoP variants in milk or blood. In humans and bovine, F2-IsoP were associated with severe OS and lipid peroxidation during exacerbated or chronic inflammation [25,26,37,38,59]. Nevertheless, differential production of A-, D-, and E-IsoP may be essential for regulation of inflammation-mediated tissue repair or possibly act as anti-inflammatory mediators. Indeed, Putman et al. [45] observed greatest A2-IsoP concentrations 2 days after cessation of lactation, whereas A1-IsoP was not detected until 12 days after involution. The lack of A- and D-IsoP in this study may be due to the unregulated inflammation and excess production of ROS compared to non-infectious and regulated inflammatory events. However, the lack of data on regulating mechanisms or bioactivity of the various IsoP limits our understanding, particularly in non-human species, and therefore additional research into IsoP production and bioactivity is essential.

## 5. Conclusions

This study highlights for the first time the importance of oxidant status and the severity of the pre-existing inflammatory state on the efficacy of FM during coliform mastitis. Additionally, we offer unique insights into potential biomarkers to determine and improve the efficacy of FM treatment for endotoxemia caused by coliform mastitis. Ability of FM to eliminate blood NEFAs and total IsoP elevations in cows that died compared to those that survived from pre- to post-treatment, as well as a reduction in milk ROS from pre- to post-FM treatment, indicate that the FM treatment was effective in reducing signs of inflammation. However, the lack of IsoP reduction in milk among cows that died, compared to the reduction in not only total IsoP but specific IsoP linked to OS by excessive inflammation, highlights the extent of the local tissue damage. Degree of blood–milk barrier compromise may therefore be crucial for survivability, and treatment with FM does not reduce inflammation sufficiently to ameliorate excessive tissue damage. Additional NSAIDs are currently being examined to improve inflammatory responses and milk production during mastitis and onset of lactation. However, inhibition of proper inflammatory function [60] and lack of effect on haptoglobin concentrations [61] further highlight the need for improved markers of inflammation and anti-inflammatory treatment. A better understanding of the inflammatory state of cows at the time of NSAID treatment has the potential to improve the clinical outcomes of severe coliform mastitis with systemic involvement.

## Figures and Tables

**Table 1:** Blood biomarkers of oxidant status over the course of systemic coliform mastitis with FM treatment in Holstein cows.

**(a) All animals**	**Concentrations**								
	**Pre-FM Treatment (*N* = 8)**			**Post-FM Treatment (*N* = 8)**			**Resolution (*N* = 7)**		
**Biomarker**	**Mean**		**SEM**	**Mean**		**SEM**	**Mean**		**SEM**
ROS (RFU/µL)	2.358 ^ab^	±	0.227	1.859 ^a^	±	0.227	2.914 ^b^	±	0.239
AOP (TE/µL)	4.415 ^a^	±	0.170	5.043 ^b^	±	0.170	4.270 ^a^	±	0.181
OSi (ROS/AOP)	0.538 ^a^	±	0.056	0.373 ^b^	±	0.056	0.716 ^c^	±	0.059
**(b) Survival only**	**Concentrations**								
	**Pre-FM Treatment (*N* = 4)**			**Post-FM Treatment (*N* = 4)**			**Resolution (*N* = 4)**		
**Biomarker**	**Mean**		**SEM**	**Mean**		**SEM**	**Mean**		**SEM**
ROS (RFU/µL)	2.528 ^a^	±	0.310	1.880 ^a^	±	0.310	2.535 ^a^	±	0.310
AOP (TE/µL)	4.532 ^ab^	±	0.224	5.335 ^a^	±	0.224	4.505 ^b^	±	0.224
OSi (ROS/AOP)	0.562 ^a^	±	0.073	0.348 ^b^	±	0.073	0.602 ^a^	±	0.073

^a–c^ Means within a row with different superscripts are different (*p* < 0.05).

**Table 2:** Milk biomarkers of oxidant status over the course of systemic coliform mastitis with FM treatment in Holstein cows.

**(a) All animals**	**Concentrations**								
	**Pre-FM Treatment (*N* = 8)**			**Post-FM Treatment (*N* = 8)**			**Resolution (*N* = 7)**		
**Biomarker**	**Mean**		**SEM**	**Mean**		**SEM**	**Mean**		**SEM**
ROS (RFU/µL)	15,221.000 ^a^	±	2985.490	7695.380 ^b^	±	2985.490	11,000.00 ^ab^	±	3066.370
AOP (TE/µL)	6.363 ^a^	±	0.900	8.563 ^b^	±	0.900	6.918 ^a^	±	0.922
OSi (ROS/AOP)	3750.410 ^a^	±	1031.890	1467.460 ^b^	±	1031.890	3031.870 ^ab^	±	1059.940
**(b) Survival only**	**Concentrations**								
	**Pre-FM Treatment (** ***N* = 4)**			**Post-FM Treatment (** ***N* = 4)**			**Resolution (*N* = 4)**		
**Biomarker**	**Mean**		**SEM**	**Mean**		**SEM**	**Mean**		**SEM**
ROS (RFU/µL)	11,757.000 ^a^	±	4339.200	7317.380 ^a^	±	4339.200	9031.750 ^a^	±	4339.200
AOP (TE/µL)	7.750 ^a^	±	0.578	11.550 ^b^	±	0.578	9.325 ^a^	±	0.578
OSi (ROS/AOP)	1556.820 ^a^	±	1056.530	646.830 ^a^	±	1056.530	984.500 ^a^	±	1056.530
**(c) Death only**	**Concentrations**								
	**Pre-FM Treatment (** ***N* = 4)**			**Post-FM Treatment (** ***N* = 4)**			**Resolution (** ***N* = 3)**		
**Biomarker**	**Mean**		**SEM**	**Mean**		**SEM**	**Mean**		**SEM**
ROS (RFU/µL)	18,685.000 ^a^	±	4339.200	8073.380 ^b^	±	4339.200	13,548.000 ^ab^	±	4566.120
AOP (TE/µL)	4.975 ^a^	±	0.578	5.575 ^a^	±	0.578	4.537 ^a^	±	0.636
OSi (ROS/AOP)	5944.010 ^a^	±	1056.530	2288.080 ^b^	±	1056.530	5542.340 ^a^	±	1111.05

^a–b^ Means within a row with different superscripts are different (*p* < 0.05).

**Table 3:** Milk biomarkers of oxidative stress over the course of systemic coliform mastitis with FM treatment in Holstein cows.

**(a) All animals**	**Concentrations**								
	**Pre-FM Treatment (*N* = 8)**			**Post-FM Treatment (*N* = 8)**			**Resolution (*N* = 7)**		
**Biomarker (nM)**	**Mean**		**SEM**	**Mean**		**SEM**	**Mean**		**SEM**
5-iso-iPF2α-VI	9.300 ^a^	±	1.3163	3.550 ^b^	±	1.3163	3.993 ^b^	±	1.3867
8,12-iso-iPF2α-VI	22.388 ^a^	±	3.1743	7.225 ^b^	±	3.1743	7.458 ^b^	±	3.3755
8-iso-15^®^-PGF2α	2.100 ^a^	±	0.364	1.600 ^a^	±	0.364	1.119 ^a^	±	0.3878
8-iso-15-keto-PGE2	3.500 ^a^	±	0.6616	1.538 ^a^	±	0.6616	2.206 ^a^	±	0.7058
Total IsoP	37.288 ^a^	±	4.769	13.913 ^b^	±	4.769	14.733 ^b^	±	5.0478
**(b) Survival only**	**Concentrations**								
	**Pre-FM Treatment (** ***N* = 4)**			**Post-FM Treatment (** ***N* = 4)**			**Resolution (** ***N* = 4)**		
**Biomarker (nM)**	**Mean**		**SEM**	**Mean**		**SEM**	**Mean**		**SEM**
5-iso-iPF2α-VI	10.775 ^a^	±	1.9483	3.400 ^b^	±	1.9483	4.525 ^ab^	±	1.9483
8,12-iso-iPF2α-VI	26.350 ^a^	±	4.6684	7.250 ^b^	±	4.6684	8.125 ^b^	±	4.6684
8-iso-15^®^-PGF2α	2.050 ^a^	±	0.5489	1.625 ^a^	±	0.5489	1.400 ^a^	±	0.5489
8-iso-15-keto-PGE2	3.300 ^a^	±	0.996	1.575 ^a^	±	0.996	1.725 ^a^	±	0.996
Total IsoP	42.475 ^a^	±	7.0826	13.850 ^b^	±	7.0826	15.775 ^b^	±	7.0826

^a–b^ Means within a row with different superscripts are different (*p* < 0.05).

## Data Availability

All data generated or analyzed during this study are included in this published article.
